# Could polyhexanide and chlorine dioxide be used as an alternative to chlorhexidine? A systematic review

**DOI:** 10.1590/1516-3180.2020.0776.R1.18052021

**Published:** 2021-12-17

**Authors:** Dayanne Simões Ferreira Santos, Mariela Peralta-Mamani, Felipe Suaki Brandão, Flaviana Bombarda Andrade, Thiago Cruvinel, Paulo Sérgio da Silva Santos

**Affiliations:** I DDS. Dentist and Master’s Student, Department of Surgery, Stomatology, Pathology and Radiology, Faculdade de Odontologia de Bauru (FOB), Universidade de São Paulo (USP), São Paulo (SP), Brazil.; II DDS, MSc. Dentist and Doctoral Student, Department of Surgery, Stomatology, Pathology and Radiology, Faculdade de Odontologia de Bauru (FOB), Universidade de São Paulo (USP), São Paulo (SP), Brazil.; III DDS, MSc. Dentist and Doctoral Student, Department of Dentistry, Dentistry School, Universidade Estadual de Maringá (UEM), Maringá (PR), Brazil.; IV DDS, PhD. Dentist and Associate Professor, Department of Dentistry, Endodontics and Dental Materials, Faculdade de Odontologia de Bauru (FOB), Universidade de São Paulo (USP), São Paulo (SP), Brazil.; V DDS, PhD. Dentist and Associate Professor, Department of Pediatric Dentistry, Orthodontics and Public Health, Faculdade de Odontologia de Bauru (FOB), Universidade de São Paulo (USP), São Paulo (SP), Brazil.; VI DDS, PhD. Dentist and Associate Professor, Department of Surgery, Stomatology, Pathology and Radiology, Faculdade de Odontologia de Bauru (FOB), Universidade de São Paulo (USP), São Paulo (SP), Brazil.

**Keywords:** Chlorhexidine, Polyhexanide [supplementary concept], Chlorine dioxide [supplementary concept], Mouthwash, Systematic review [publication type], Oral infection control, Mouth rinses, Oral hygiene, Adverse effects, Dental management

## Abstract

**BACKGROUND:**

Maintenance of oral microbiota balance is the simplest way to prevent infectious oral diseases, through controlling dental biofilm. Combined use of mouthwash and mechanical removal has been shown to be a very effective way for this.

**OBJECTIVES:**

To identify clinical studies comparing the antimicrobial effect and possible adverse effects and/or side effects of chlorhexidine-based mouthwashes with those of mouthwashes containing chlorine dioxide and/or polyhexanide, for controlling oral microbiota.

**DESIGN AND SETTING:**

Systematic review designed by the stomatology sector of postgraduation in applied dental sciences of Bauru Dentistry School, University of São Paulo, Brazil.

**METHODS:**

A systematic review was conducted using online databases (PubMed, Embase, Web of Science and Science Direct) up to April 8, 2020. The search was conducted using the Preferred Reporting Items for Systematic Reviews and Meta-Analyses (PRISMA) guidelines.

**RESULTS:**

The studies included comprised eight articles published between 2001 and 2017. A total of 295 young adults, adults and elderly people were evaluated (males 44.75% and females 55.25%). Three articles compared polyhexanide with chlorhexidine and five articles compared chlorine dioxide with chlorhexidine. No studies comparing all three mouthwashes were found. The concentrations of the study solutions were quite varied, and all rinses had an antimicrobial effect. In four studies, it was stated that no side effects or adverse effects had been found. Three studies did not address these results and only one study addressed side effects and/or adverse effects.

**CONCLUSION:**

Mouthwashes containing chlorine dioxide and polyhexanide are viable alternatives to chlorhexidine, since they reduce oral biofilm and have little or no reported side or adverse effects.

## INTRODUCTION

### Rationale

Control of dental biofilm and maintenance of the balance of the oral microbiota is the simplest way to prevent diseases such as periodontal disease and dental caries.^1^ Combined use of mouthwash and mechanical removal has been shown to be a very effective way for controlling cariogenic and periodontogenic biofilms.^2^ These biofilms may present a risk of systemic dissemination through microaspiration or the hematogenous route, with consequent secondary infections.

Among the various chemical agents used to control dental biofilms, chlorhexidine (CHX) is the gold standard because of its excellent bacteriostasis, substantivity, non-specificity and broad spectrum.^3^^,^^4^ However, there is evidence that prolonged use of CHX has adverse effects, such as tooth and restoration staining, mucosal irritation, microbial resistance and changes to taste sensation, thus restricting its use to specific cases in dentistry.^3^^,^^4^^,^^5^^,^^6^

Polyhexamethylene biguanide or polyhexanide (PHMB) and chlorine dioxide (ClO2) are alternatives to CHX. Studies have demonstrated that PHMB has a broad antimicrobial spectrum, low risk of contact hypersensitivity and good tolerability by cells and tissues, and that it also promotes wound healing.^7^^,^^8^ Interestingly ClO2 is not particularly influenced by variations in mouth pH after activation. ClO2 has action against bacteria, viruses and fungi and high water solubility that provides it with the ability to penetrate the biofilm quickly to exert its action.^9^^,^^10^ To our knowledge, there have not been any clinical or in vitro studies aimed at comparing the effects of these three solutions (CHX, PHMB and ClO2).

## OBJECTIVES

The aim of this systematic review was to identify clinical studies that compared the antimicrobial effect and possible adverse and/or side effects of CHX-based mouthwashes with those of mouthwashes containing ClO2 and/or PHMB, for controlling dental biofilm.

### Research question

Two research questions were formulated:

- Do mouthwashes containing PHMB and/or ClO2 have antimicrobial efficacy in the oral microbiota comparable to that of CHX?
- Do studies with mouthwashes containing PHMB and/or ClO2 show adverse and/or side effects, in comparison with to the effects associated with CHX?

## METHODS

### Study design

This systematic review was conducted in accordance with the PRISMA guidelines (Preferred Reporting Items of Systematic Reviews and Meta-Analyses).^9^

### Participants, interventions and comparators

All the studies selected met the criteria established through the PICO strategy: (1) Participants: oral microbiota; (2) Intervention: PHMB and/or ClO2; (3) Control: CHX; and (4) Outcomes: antimicrobial efficacy of mouthwashes containing PHMB and/or ClO2, compared with that of CHX.

### Systematic review protocol

The protocol for this systematic review was registered in PROSPERO (CRD42019115929) and is available on the website www.crd.york.ac.uk/PROSPERO/.

### Search strategy

A search of the literature was conducted to survey clinical studies that aimed to investigate the antimicrobial action of mouthwashes containing PHMB and ClO2, compared with that of CHX. The studies included were identified based on a search strategy for each electronic database: PubMed, EMBASE, Web of Science and Science Direct. The search strategy was designed with Boolean operators (AND/OR) to identify all studies on this topic published in English, Portuguese or Spanish up to December 21, 2020. The descriptors used were “Chlorhexidine”, “Polyhexanide”, “Dioxide Chlorine” and “Mouthwash”. The search strategies are detailed in Table 1.

In the Science Direct database, filters for research articles (31) and conference abstracts (2) were activated in order to exclude texts from encyclopedias, book chapters and other sources.

**Table 1.** Databases and search strategy

Database	Search Strategy
PubMed	((((“chlorhexidine”[MeSH Terms] OR “chlorhexidine”[All Fields]) AND (“mouthwashes”[Pharmacological Action] OR “mouthwashes”[MeSH Terms] OR “mouthwashes”[All Fields] OR “mouthwash”[All Fields])) OR (“chlorhexidine”[MeSH Terms] OR “chlorhexidine”[All Fields]) OR (“chlorhexidine gluconate”[Supplementary Concept] OR “chlorhexidine gluconate”[All Fields])) AND (phmb[All Fields] OR (“polihexanide”[Supplementary Concept] OR “polihexanide”[All Fields] OR “polyhexamethylene biguanide”[All Fields]) OR (“polihexanide”[Supplementary Concept] OR “polihexanide”[All Fields] OR “polyhexamethylenbiguanid”[All Fields]) OR (“polihexanide”[Supplementary Concept] OR “polihexanide”[All Fields]) OR (dioxide[All Fields] AND (“chlorine”[MeSH Terms] OR “chlorine”[All Fields])) OR (“chlorine dioxide”[Supplementary Concept] OR “chlorine dioxide”[All Fields])) AND (mouthrinse[All Fields] OR (“mouthwashes”[Pharmacological Action] OR “mouthwashes”[MeSH Terms] OR “mouthwashes”[All Fields] OR “mouthwash”[All Fields]) OR (“mouthwashes”[Pharmacological Action] OR “mouthwashes”[MeSH Terms] OR “mouthwashes”[All Fields]) OR (“mouthwashes”[Pharmacological Action] OR “mouthwashes”[MeSH Terms] OR “mouthwashes”[All Fields] OR (“mouth”[All Fields] AND “bath”[All Fields]) OR “mouth bath”[All Fields]) OR (“mouthwashes”[Pharmacological Action] OR “mouthwashes”[MeSH Terms] OR “mouthwashes”[All Fields] OR (“mouth”[All Fields] AND “rinse”[All Fields]) OR “mouth rinse”[All Fields]) OR (“mouthwashes”[Pharmacological Action] OR “mouthwashes”[MeSH Terms] OR “mouthwashes”[All Fields] OR (“mouth”[All Fields] AND “wash”[All Fields]) OR “mouth wash”[All Fields]) OR (“mouthwashes”[Pharmacological Action] OR “mouthwashes”[MeSH Terms] OR “mouthwashes”[All Fields] OR (“bath”[All Fields] AND “mouth”[All Fields])) OR (“mouthwashes”[Pharmacological Action] OR “mouthwashes”[MeSH Terms] OR “mouthwashes”[All Fields] OR (“baths”[All Fields] AND “mouth”[All Fields])) OR (“mouthwashes”[Pharmacological Action] OR “mouthwashes”[MeSH Terms] OR “mouthwashes”[All Fields] OR (“mouth”[All Fields] AND “baths”[All Fields]) OR “mouth baths”[All Fields]) OR (“mouthwashes”[Pharmacological Action] OR “mouthwashes”[MeSH Terms] OR “mouthwashes”[All Fields] OR (“mouth”[All Fields] AND “rinses”[All Fields]) OR “mouth rinses”[All Fields]) OR (“mouthwashes”[Pharmacological Action] OR “mouthwashes”[MeSH Terms] OR “mouthwashes”[All Fields] OR (“rinse”[All Fields] AND “mouth”[All Fields])) OR (“mouthwashes”[Pharmacological Action] OR “mouthwashes”[MeSH Terms] OR “mouthwashes”[All Fields] OR (“rinses”[All Fields] AND “mouth”[All Fields])) OR (“mouthwashes”[Pharmacological Action] OR “mouthwashes”[MeSH Terms] OR “mouthwashes”[All Fields] OR (“wash”[All Fields] AND “mouth”[All Fields]))))
Embase	((‘chlorhexidine mouthwash’/exp OR ‘chlorhexidine mouthwash’ OR ((‘chlorhexidine’/exp OR chlorhexidine) AND (‘mouthwash’/exp OR mouthwash)) OR ‘chlorhexidine’/exp OR chlorhexidine OR ‘chlorhexidine gluconate’/exp OR ‘chlorhexidine gluconate’ OR ((‘chlorhexidine’/exp OR chlorhexidine) AND (‘gluconate’/exp OR gluconate))) AND (phmb OR ‘polyhexamethylene biguanide’/exp OR ‘polyhexamethylene biguanide’ OR (polyhexamethylene AND (‘biguanide’/exp OR biguanide)) OR polyhexamethylenbiguanid OR ‘polihexanide’/exp OR polihexanide OR ‘dioxide chlorine’ OR ((‘dioxide’/exp OR dioxide) AND (‘chlorine’/exp OR chlorine)) OR ‘chlorine dioxide’/exp OR ‘chlorine dioxide’ OR ((‘chlorine’/exp OR chlorine) AND (‘dioxide’/exp OR dioxide))) AND (‘mouthrinse’/exp OR mouthrinse OR ‘mouthwash’/exp OR mouthwash OR ‘mouthwashes’/exp OR mouthwashes OR ‘mouth bath’ OR ((‘mouth’/exp OR mouth) AND (‘bath’/exp OR bath)) OR ‘mouth rinse’/exp OR ‘mouth rinse’ OR ((‘mouth’/exp OR mouth) AND rinse) OR ‘mouth wash’/exp OR ‘mouth wash’ OR ((‘mouth’/exp OR mouth) AND wash) OR ‘bath, mouth’ OR (bath, AND (‘mouth’/exp OR mouth)) OR ‘baths, mouth’ OR (baths, AND (‘mouth’/exp OR mouth)) OR ‘mouth baths’ OR ((‘mouth’/exp OR mouth) AND (‘baths’/exp OR baths)) OR ‘mouth rinses’/exp OR ‘mouth rinses’ OR ((‘mouth’/exp OR mouth) AND rinses) OR ‘rinse, mouth’ OR (rinse, AND (‘mouth’/exp OR mouth)) OR ‘rinses, mouth’ OR (rinses, AND (‘mouth’/exp OR mouth)) OR ‘wash, mouth’ OR (wash, AND (‘mouth’/exp OR mouth))))
Web of Science	((((Chlorhexidine mouthwash OR Chlorhexidine) OR Chlorhexidine gluconate) AND (((((phmb OR polyhexamethylene biguanide) OR polyhexamethylenbiguanide) OR polihexanide) OR dioxide chlorine) OR chlorine dioxide)) AND ((((((((((((mouthrinse OR mouthwash) OR mouthwashes) OR Mouth Bath) OR Mouth Rinse) OR Mouth Wash) OR Bath, Mouth) OR Baths, Mouth) OR Mouth Baths) OR Mouth Rinses) OR Rinse, Mouth) OR Rinses, Mouth) OR Wash, Mouth)).
Science Direct	(Chlorhexidine OR Chlorhexidine gluconate) AND (phmb OR polyhexamethylene biguanide OR polyhexamethylen biguanid OR polihexanide OR chlorine dioxide) AND (mouthrinse OR mouthwash OR mouthwashes OR Mouth Bath OR Mouth Rinse OR Mouth Wash)

### Eligibility criteria

This review included clinical studies that evaluated the effectiveness of mouthwashes and studies that compared the action of PHMB and/or ClO2 in relation to CHX, regardless of the participants’ age, sex, systemic changes or medication use.

The following types of studies were excluded: literature review articles, clinical cases or case series, studies that did not evaluate mouthwashes, studies not related to dentistry, in vitro, in situ and animal studies and studies published in other languages.

### Data sources, study selection and data extraction

All records collected were moved to a folder of the reference manager EndNote Web (www.myendnoteweb.com). Any duplication of references was identified and then deleted.

Studies were identified independently by two reviewers (D.S.F.S. and F.S.B.) in two phases: 1. Reading the titles and summaries of each article; and 2. Reading the full text. Any discrepancies during either of these phases were resolved through discussion with a third reviewer (P.S.S.S.).

All studies included were independently examined by two reviewers (D.S.F.S. and F.S.B.) and their main characteristics were extracted in order to perform data synthesis and study quality assessment. Only the information described in the articles was considered.

### Data analysis

A narrative data synthesis was carried out, structured around the characteristics of each study, i.e. the microbiological count, type of microorganism, characteristics of the population, parameters evaluated and results obtained.

### Risk of bias

Two reviewers (D.S.F.S. and F.S.B.) independently assessed the risk of bias in the studies included through using the Cochrane risk of bias tool (RoB 2.0, 2008), which is available in the Cochrane manual for developing systematic intervention reviews, version 5.1.0 (Cochrane Handbook, Oxford, United Kingdom, and Melbourne, Australia).^10^ Any discrepancies were resolved by a third reviewer (P.S.S.S.). This tool was chosen to assess the risk of bias in randomized clinical trials^10^ in terms of seven domains: generation of random sequence, allocation concealment, blinding of participants and professionals, blinding of outcome evaluators, incomplete outcomes, selective outcome report and others. These were classified as presenting “low risk”, “high risk” or “uncertain risk”, in accordance with each criterion of the tool.^10^ Afterwards, the data were inserted into the Review Manager (RevMan Version 5.3, Cochrane Manager Review Center, Oxford, United Kingdom) software, and a risk-of-bias graph was generated.

## RESULTS

### Study selection

**Figure 1.**
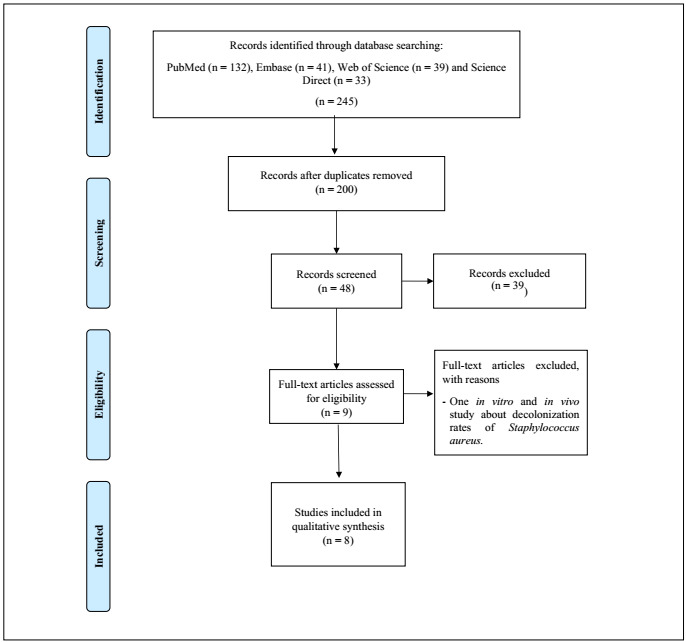
Flow diagram of the studies included for the review.

A total of 245 studies were initially identified in the following databases: PubMed (n = 132), Embase (n = 41), Web of Science (n = 39) and Science Direct (n = 33). Thirty studies were excluded due to duplication. Among the remainder, 48 studies were selected for reading the title and abstract and 39 of these were excluded for the following reasons: they were in vitro or in vivo studies, did not use CHX as a control (comparison), did not use PHMB and/or ClO2 as an intervention or did not use mouthwashes. Thus, the full texts of nine studies were read. From this, one further study were excluded because it did not meet the eligibility criteria (it was an in vitro and in vivo study about decolonization rates of Staphylococcus aureus). Hence, the final analysis was conducted on eight studies. The detailed sequence can be seen in the study selection flowchart^9^ (Figure 1).

### Study characteristics

Table 1 shows the general characteristics of the studies included, which were published between 2001 and 2017. The search was carried out without restriction on publication date. These studies were conducted in Europe and Asia (Germany, Switzerland, Turkey, India and Indonesia). All of them were randomized clinical studies, and microbiological analyses were performed.^8^^,^^11^^,^^12^^,^^13^^,^^14^^,^^15^^,^^16^^,^^17^ In total, 295 individuals were evaluated and, in the studies in which the participants were separated according to sex,^8^^,^^11^^.,^^12^^,^^13^^,^^14^^,^^15^^,^^16^^,^^17^ 44.75% were men and 55.25% women. Six studies involved young adults with an average age between 18 and 25 years, ^8^^,^^11^^,^^12^^,^^13^^,^^15^^,^^17^^,^^18^ one involved adults and the elderly^14^ with a mean age of 60.8 ± 15.0 years and one involved adolescents aged 11-16 years.^16^

### Evaluation profile of the clinical trials

One study evaluated the antifungal effects of ClO2 compared with those of CHX;^14^ one compared ClO2 with CHX, against the chromogenic bacterium species Actinomyces;^16^ three evaluated the effect of PHMB compared with CHX, on oral biofilm;^11^^,^^12^^,^^13^ and three compared the effects of ClO2 with those of CHX, on oral biofilm.^8^^,^^15^^,^^17^^,^^18^

One study evaluated totally edentulous individuals and their dentures,^14^ six evaluated the teeth and mucous membranes of young adults,^8^^,^^11^^,^^12^^,^^13^^,^^15^^,^^17^ one evaluated the tongue coating,^8^ one evaluated individuals who had undergone orthodontic treatment^17^ and one evaluated molar dental sulcus pigmentation in children.^16^

### Mouthwashes

Three studies compared PHMB with CHX^11^^,^^12^^,^^13^ and the other five compared ClO2 with CHX.^8^^,^^14^^,^^15^^,^^16^^,^^17^ No studies comparing PHMB with ClO2 or all three solutions simultaneously were found. The CHX concentration that was most used was 0.20%,^8^^,^^14^^,^^15^ followed by 0.12%^11^^,^^12^ and 0.1%.^16^ The PHMB concentrations used were 0.04%,^11^ 0.12%^12^ and 0.20%^13^ and those of ClO2 were 0.01%,^15^ 0.80%^14^ and 0.1%.^16^

In all studies,^8^^,^^11^^,^^16^^,^^20^ the frequency of use was two washes per day, i.e. one in the morning and other at night, for each mouthwash. In addition to differences in concentrations, there were differences in quantity, duration of exposure to mouthwash solution and duration of the study (Table 2). The study that evaluated totally edentulous individuals^14^ gave the recommendation that individuals should immerse their dentures in the mouthwash, overnight for 15 days.

**Table 2.** Summary of information contained in the articles included in this review

Author, country	Number of individuals/sex/mean age	Mouthwash	Concentration		Quantity (ml)/duration of exposure (s)	Duration of study (days)	What was studied	Microorganism - Microbiological count	Conclusion
Rosin et al.,^11^ Germany	16/12 men/4 women/23.4 years	PHMB (0.04%) mouth rinse: 0.2% Lavasept (Fresenius Kabi, Bad Homburg, Germany), 0.1% aromatic oil (Henkel, Düsseldorf, Germany), 0.1% Cremophor (Henkel, Düsseldorf, Germany), 10.4% ethanol (96%), 90.2% Ringer’s solution. Placebo mouth rinse: 0.1% aromatic oil, 0.1% Cremophor, 10.4% ethanol (96%), 90.4% deionized water. CHX mouth rinse (0.12%): 6% chlorhexidine digluconate (20%) (Henkel, Düsseldorf, Germany), 94% deionized water. Skinsept mucosa (diluted in 0.12% chlorhexidine): 40% SkinseptA mucosa (Henkel, Düsseldorf, Germany), 6.24% ethanol (96%), 1% hydrogen peroxide (30.42%), 0.12% lactic acid, 52.64% deionized water.	CHX - 0.12%	PHMB - 0.04%	20/60	4	The effects on dental biofilm and oral bacterial count were compared.	Oral biofilm - Dental biofilm index and smears of dental surface and cheek mucosa (on days 1 and 5) and CFU count per sample. Four hours after the first use of mouthwashes, there was no statistical difference between PHMB, Skinsept and placebo, while CHX was superior for destruction of dental biofilm after 4 hours. In the mucosa, 4 hours after the first use of the mouthwashes, all mouthwashes were more effective than placebo for destroying oral biofilm. Twelve hours after the final use of mouthwashes, CHX was the most effective in destroying oral biofilm, PHMB was statistically more effective than placebo, while Skinsept did not show any difference in reducing biofilm, compared with placebo.	CHX 0.12% was more effective than PHMB 0.04% and placebo for destroying bacterial biofilm. The substantivity of CHX was always 12 hours. The substantivity of PHMB was 4 hours in the oral mucosa only. The antibacterial effect of PHMB was significantly greater than placebo on the mucosa alone.
Rosin et al.,^12^ Germany	16/ 6 men/ 10 women/ 23.4 years	PHMB (0.12%) mouth rinse: 0.6% LavaseptA (Fresenius Kabi, Bad Homburg, Germany), 0.1% aromatic oil (Henkel, Düsseldorf, Germany), 0.1% Cremophor (Henkel, Düsseldorf, Germany), 10.4% ethanol (96%), 88.8% Ringer’s solution. Placebo mouth rinse: 0.1% aromatic oil (Henkel, Düsseldorf, Germany), 0.1% Cremophor (Henkel, Düsseldorf, Germany), 10.4% ethanol (96%), 89.4% deionized water. CHX mouth rinse (0.12%): 0.6% chlorhexidine digluconate (20%) (Henkel, Düsseldorf, Germany), 94% deionized water. Essential oil mouth rinse: Listerine antiseptic (Warner-Lambert, Consumer Healthcare Products, Freiburg, Germany).	CHX - 0.12%	PHMB - 0.12%	20/60	4	To increase the PHMB concentration from 0.04% to 0.12% and evaluate the effects on the biofilm formed and oral bacterial counts, compared with CHX 0.12%. To include an established commercial product (Listerine) available for another comparison.	Oral biofilm - Dental biofilm index and smears of the dental surface and cheek mucosa (on days 1 and 5) and CFU count per sample. Four hours after the first use of mouthwashes, no statistical difference was observed between PHMB and Listerine or PHMB and CHX regarding destruction of dental biofilm; and 12 hours after the final use of mouthwashes, PHMB was more effective for inhibiting bacterial growth than Listerine. In the mucosa, 4 hours after the first use of mouthwashes, CHX was the most effective for destruction of oral biofilm. Twelve hours after the final use of rinses, CHX was the most effective and PHMB was significantly better than placebo for destroying oral biofilm.	PHMB mouthwash showed significantly greater inhibition of bacterial biofilm growth than placebo. The bacterial count indicated persistence of PHMB antimicrobial activity 4 hours after use.
Welk et al.,^13^ Germany	16/ 6 men/ 10 women/ 21.1 years	PHMB (0.2%) mouth rinse: 1.0% Lavasept s containing 20% PHMB (Fresenius Kabi, Bad Homburg, Germany), 0.1% aromatic oil (Henkel, Düsseldorf, Germany), 0.1% Cremophor (Henkel), 10.4% ethanol (96%), 88.6% Ringer’s solution. CHX (0.12%) mouth rinse: 6% solution of 20% chlorhexidine digluconate stock solution (Henkel), 94% deionized water. Triclosan (0.3%)/ copolymer (2.0%) mouth rinse: Commercially available Colgate Total Plax s mouth rinse (Colgate-Palmolive Company, New York, NY, United States) containing 0.3% 2,4,40-trichloro-20-hydroxydiphenyl ether/2.0% polyvinyl methyl ether maleic acid (PVM/MA) copolymer. Placebo mouth rinse: 0.1% aromatic oil (Henkel), 0.1% Cremophor (Henkel), 10.4% ethanol (96%), 89.4% deionized water.	CHX - 0.12%	PHMB - 0.20%	20/60	5	Comparison of mouthwash containing PHMB (0.2%) with mouthwash containing CHX (0.12%), to evaluate its effect on the growth of dental biofilm and on oral bacterial count.	Oral biofilm - Quigley & Hein^18^ dental biofilm index (QHI), as modified by Turesky et al.^19^ After the first use of mouthwashes, it was observed that CHX was statistically more effective in destroying dental biofilm than other mouthwashes and 8 hours after the final use of mouthwashes, PHMB inhibited bacterial growth more effectively compared with triclosan and placebo. In the mucosa, after the first use of mouthwashes, all mouthwashes were more effective than placebo in destroying oral biofilm, but there was no statistical difference between them. Eight hours after the final use of rinses, PHMB was equally effective in destroying oral biofilm, compared with CHX.	The mouthwash with 2.0% PHMB was significantly less effective in destroying bacterial biofilm than 0.12% aqueous CHX. After 8 hours of using PHMB, inhibition of bacterial growth was still observed.
Paraskevas et al.,^15^ Switzerland	77/ 34 men/ 43 women/ 23.2 years	10 Quist-forte (containing 100-ppm free ClO2): The rinse was activated when 5 ml base solution was mixed with 5 ml activator solution. De Witte Tanden Winkel, Rotterdam, Netherlands. Corsodyl (containing 0.20% CHX, digluconate, ethanol, polyoxyl hydrogenated castor oil, sorbitol, E-125, purified water), GlaxoSmithKline, Zeist, Netherlands.	CHX - 0.20%	ClO2 - 0.01%	10/60	3	To evaluate inhibition of growth of dental biofilm through use of mouthwash containing ClO2, compared with mouthwash with CHX, over the course of a 3-day dental biofilm growth model.	Dental biofilm - Dental biofilm index - In the control group (CHX), the overall average dental biofilm index was 1.39, compared with 1.96 in the test group (ClO2), (P < 0.001).	The ClO2 rinse was a less potent bacterial biofilm inhibitor than the CHX rinse.
Uludamar et al.,^14^ Turkey	60/ 23 men/ 37 women/ 60.8 ± 15 years	Tissue conditioner material Visco-gel, Dentsply Detrey GmbH, Detrey-straße 1, D-78467 Konstanz, Germany ClO2 (0.8%) dioxidant, Frontier Pharmaceutical, Inc., Melville, NY, United States). Corsodyl (0.2% CHX gluconate), Group Laboratories SA (Pty) Ltd., Epping Industrial 1, Cape Town, South Africa.	CHX - 0.20%	ClO2 - 0.80%	30/60	15	The effect of tissue conditioning and two mouthwashes on resolution of clinical symptoms of prosthetic stomatitis and on reduction of Candida albicans.	Candida albicans - The method of Budtz-Jorgensen et al.^20^ was used to classify the clinical effects of the treatment: Healing (without inflammation) - tissue conditioner: 40%; ClO2: 60% and CHX: 70%. Improvement (decrease in inflammation) - tissue conditioner: 25%; ClO2: 25% and CHX: 20%. Failure (no change in inflammation) - tissue conditioner: 35%; ClO2: 15% and CHX: 10%. The UFC/ml count was used to assess the effect on fungal biofilm - before/after UFC treatment/ml (P-value): tissue conditioner: 208.35/196.15 (P = 0.4); ClO2: 204.75/74.21 (P = 0.001) and CHX: 202.24/57.81 (P = 0.001).	Use of both mouthwashes (ClO2 and CHX) eliminated hyphae, decreased palatal inflammation and eliminated Candida colonization.
Yadav et al.,^8^ India	25/ 11 men/ 14 women/ 19.8 years	Stabilized ClO2 mouth rinse in aqueous vehicle Fresh Chlor (Rowpar Group Pharmaceuticals, Bangalore, India). CHX (0.2%) gluconate mouth rinse in aqueous vehicle Hexedine (ICPA, Bangalore, India).	CHX - 0.20%	ClO2 - ur	10/60	5	To evaluate the effectiveness of a mouthwash containing stabilized ClO2 and a mouthwash containing CHX for inhibiting accumulation of biofilm on the tongue and formation of dental biofilm.	Oral biofilm - The marine dental biofilm index as modified by Rustogi was used to evaluate the teeth, the Winkel index and wet weight of the coating were used to evaluate the tongue and microbiological analysis was done using UFC. on samples collected from the dental and mucosal surfaces. The marine biofilm index as modified by Rustogi, the Winkel index and the wet weight of the tongue coating did not show any statistical difference between the groups. The biofilm collected after 4 hours demonstrated that use of CHX gave rise to UFC/sample smaller than what resulted from use of ClO2 on the teeth: mean CHX 30.6800 and ClO2: 35.8800 (P = 0.001); and on the mucosa: mean CHX: 37.6400 and ClO2: 45.2800 (P = 0.00 (6.244E-5)).	The inhibitory properties against dental biofilm, the rate of accumulation of tongue biofilm and the antibacterial properties of the mouthwash with ClO2 were comparable to those of the mouthwash with CHX.
Yeturu et al.,^17^ India	85/ 40 men/ 45 women Aloe vera group (21.53 ± 3.41); CHX group (21.72 ± 4.67) and ClO2 group (21.70 ± 3.01).	Aloe vera, ClO2 and CHX.	CHX - ur	ClO2 - ur	10/60	15	To evaluate the effect of mouthwashes containing Aloe vera, ClO2 and CHX on biofilm and gingivitis during orthodontic treatment.	Dental biofilm - Dental biofilm index of Silness and Loe and gingival index. Average percentage reduction in the dental biofilm index: Aloe vera (20.38%), CHX (31.59%) and ClO2 (30.29%), with P = 0.03. Average percentage reduction in the gingival index: Aloe vera (9.88%), CHX (16.30%) and ClO2 (12.22%), with P = 0.04.	Aloe vera and ClO2 showed reductions in dental and gingival biofilm rates that were almost the same as that of CHX over a period of 15 days. Therefore, ClO2 and Aloe vera may be suitable and economical alternatives to CHX.
Eunike et al.,^16^ Indonesia	16/ ur/ 6-11 years (age variation)	Mouthwash containing ClO2 (0.1%) and mouthwash containing CHX (0.1%).	CHX - 0.10%	ClO2 - 0.10%	10/30	7	To evaluate the antibacterial effects of mouthwashes on the bacterial viability of Actinomyces sp. as a black spot agent.	Actinomyces sp. - Feasibility test with MTT test and culturing of black spot samples by means of visual inspection and Gram staining. Average viability (from optical density) of Actinomyces before/after using rinses (P-value) was: CHX: 0.67/0.54 (P = 0.01) and ClO2: 0.73/0.40 (P = 0.001).	Mouthwash containing 0.1% ClO2 had greater antibacterial effect against Actinomyces sp. than rinse containing 0.1% CHX.

### Study outcomes

The primary outcome from this systematic review was to report on the antimicrobial efficacy of mouthwashes containing PHMB and/or ClO2, compared with those containing CHX. The secondary outcome was to report on the adverse effects of mouthwashes.

### Antimicrobial efficacy of mouthwashes

All three studies that compared PHMB with CHX used a concentration of 0.12% for CHX. These studies evaluated the action of mouthwashes on bacteria. Among their conclusions, one was that the substantivity of CHX was always 12 hours.^11^^,^^12^^,^^13^

Regarding the biofilm index, the studies showed that there were significantly lower rates with CHX than with PHMB 0.04% (P = 0.038).^11^ There were no statistically significant differences between PHMB 0.12% and CHX (P > 0.05),^12^ and PHMB 0.2% was significantly less effective on the biofilm index than CHX (P = 0.016).^13^

The bacterial count was investigated at two times: four hours after using the mouthwash and five days after this. Evaluation of the bacterial count of the dental surface showed that CHX was significantly more effective in reducing the bacterial count than PHMB 0.04%, at both times evaluated (four hours, P = 0.003; five days, P = 0.030).^11^ There was no statistically significant difference between PHMB 0.12% and CHX (P = 0.085) after four hours, while after five days of use, PHMB 0.12% was significantly less effective than CHX (P = 0.008).^12^ In the first four hours, with PHMB 0.20%, there was no significant difference compared with CHX (P = 0.623); after five days of use, PHMB 0.2% significantly inhibited bacterial growth, compared with CHX (P = 0.029).^13^

Evaluation of bacterial counts on the mucosal surface showed that CHX was significantly more effective than PHMB 0.04% (P = 0.42)^11^ and PHMB 0.12% (P = 0.013)^12^ after the first four hours and after five days of using PHMB 0.04% (P = 0.007)^11^ and PHMB 0.12% (P = 0.000).^12^ There were no significant differences between PHMB 0.2% and CHX four hours after use (P = 0.738) or five days afterwards: both solutions were equally effective (P = 1.000).^13^

Other studies compared ClO2 with CHX^8^^,^^14^^,^^15^^,^^16^^,^^17^ and found that CHX 0.2% inhibits biofilm more powerfully than ClO2 0.01% (P < 0.001).^15^ Four hours after use, CHX 0.2% was found to have been more efficient than ClO2, such that there were fewer colony-forming units (CFUs) on the mucosa (P < 0.001) and on the dental surface (P = 0.01).^8^ Regarding the biofilm index (P = 0.05), rate of accumulation of tongue biofilm (P = 0.238), presence of bacterial CFUs on the fifth day of mouthwash and application of mouthwashes for 15 days, use of ClO2 was equal to use of CHX 0.2% (P = 0.160).^8^ It was concluded that the reductions in the dental biofilm index (from 1.30 to 0.84; P < 0.01) and gingival index (from 1.43 to 1.23, P < 0.01) through use of ClO2 were similar to what was seen regarding the dental biofilm index (from 1.27 to 0.83; P < 0.01) and gingival index (from 1.63 to 1.35; P < 0.01) in a mouthwash with CHX.^17^ In evaluations on fungus, it was concluded that both rinses (ClO2 0.80% and CHX 0.20%) eliminated Candida albicans hyphae (ClO2, P = 0.03; and CHX, P > 0.01), decreased palatal inflammation (ClO2, P = 0.001; and CHX, P = 0.04) and eliminated Candida colonization (P = 0.001 for both).^14^ A single study showed that ClO2 0.1% had a greater antibacterial effect (P = 0.001) than CHX 0.1% (P = 0.01).^16^

### Adverse effects/side effects

The authors of seven studies^8^^,^^11^^,^^12^^,^^13^^,^^14^^,^^16^^,^^17^ did not mention the expected adverse or side effects: among these, the authors of four studies reported that they did not observe any adverse effects and/or side effects during their investigations,^11^^,^^12^^,^^13^^,^^14^ while such effects were not reported in the results from three studies.^8^^,^^16^^,^^17^

In one other study,^15^ a questionnaire regarding the perception of mouthwashes was applied. The participants in that study preferred the taste of ClO2 over that of CHX (P < 0.001) and reported that there was less change in taste when using ClO2 than when using CHX (P < 0.001). The taste of CHX remained in the mouth longer than that of ClO2 (P < 0.001), while use of CHX was more convenient than use of ClO2 (P < 0.001) and the perception of plaque reduction through using CHX was greater than through using ClO2 (P < 0.001).^15^

### Risk of bias

In the present study, the Cochrane risk of bias (RoB) tool^10^ was applied to assess the risk of bias in the eight randomized controlled trials that were included. The risk of bias was explored in seven domains.

**Figure 2.**
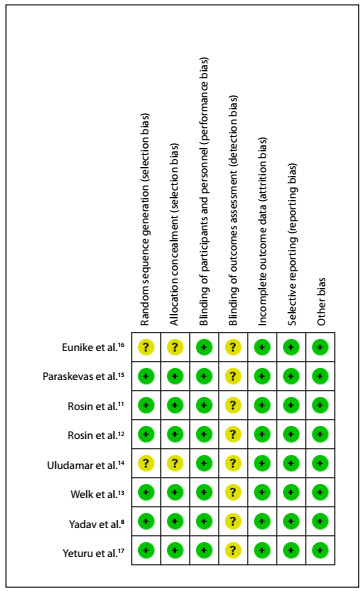
Assessment of the risk of bias in the included studies.

**Figure 3.**
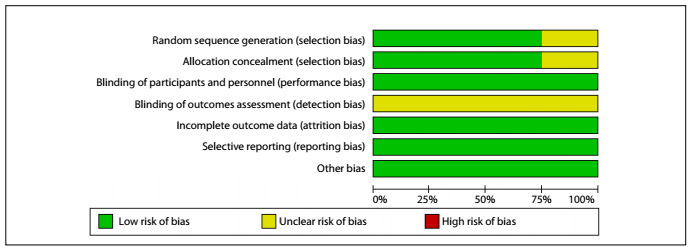
Percentages of risk of bias in studies included.

Two studies were classified as presenting an uncertain risk of bias in three domains, specifically those relating to selection bias (random sequence generation and allocation concealment) and detection bias (blinding of outcome assessment),^14^^,^^16^ given that in these studies the randomization and allocation methods were not mentioned and it was not reported whether the results were obtained through blind analysis. Six studies were classified as presenting an uncertain risk of bias in relation to detection bias (blinding of outcome assessment),^8^^,^^11^^,^^12^^,^^13^^,^^15^^,^^17^ given that it was not addressed whether blinding had been applied in order to obtain the results. The other domains of all studies were classified as having low risk of bias. No study was classified as having a high risk of bias in any domain (Figure 2). Therefore, overall, the studies included in this systematic review showed good methodological quality (Figure 3).

## DISCUSSION

Finding a mouthwash that is as effective as CHX and which has fewer adverse effects has been a challenge for researchers. In this systematic review, it was seen that in a study that compared PHMB with CHX, the residual antimicrobial action (substantivity) of PHMB^12^^,^^13^ and its antimicrobial activity were equal to those of CHX. These results make PHMB a viable alternative to CHX^12^^,^^13^ for clinical practice, considering that substantivity is a characteristic that ensures that the product continues to act after its application. All the studies included in this review that compared PHMB with CHX stated the CHX showed substantivity of 12 hours.^11^^,^^12^^,^^13^ Previous studies demonstrated that CHX showed substantivity for varying times,^21^^,^^22^^,^^23^^,^^24^^,^^25^, viz. up to 7 hours in a 2010 in vivo study,^24^ up to 24 hours in a 1974 study^22^ and up to 12 weeks in a 2009 review.^23^

In biofilm collected from the mouths of individuals to compare ClO2 with CHX, used twice a day for three days, it was found in one study^15^ that there were significant reductions in the total biofilm index in both the test (ClO2) and the control (CHX) groups, and that this reduction was observed in both groups in assessments on different surfaces, i.e. mucous membranes, teeth and upper and lower jaws. In another study,^8^ it was demonstrated that after four days, there was no statistical difference in the degree of destruction of bacteria between the two rinses,^8^ thus also showing that the antimicrobial action of ClO2 was comparable to that of CHX. In an in vitro study^26^ that was carried out to evaluate the action of ClO2 on the dental canal compared with the action of CHX, it was demonstrated that ClO2 was significantly more effective in reducing intracanal bacteria than CHX. In another randomized clinical study^27^ comparing ClO2 with sodium chloride to treat halitosis, ClO2 reduced the amount of tongue coating and Gram-positive and Gram-negative bacteria in the saliva.^27^ In dental black spots caused by Actinomyces sp., ClO2 proved to be statistically more effective in reducing the bacterial viability of Actinomyces sp. than CHX, after seven days of use.^16^ ClO2 is believed to be an effective alternative for use among children, given that this solution is not carcinogenic or allergenic and does not cause any change in taste sensation. Moreover, there are studies that have suggested that it is less toxic to humans than CHX.^16^^,^^28^ Therefore, although CHX is typically considered to be the gold standard, ClO2 is also effective for biofilm control.

When rinses containing ClO2 and CHX were applied to patients with orthodontic appliances, no statistical differences regarding reduction of the gingival index or total visible biofilm index were observed.^17^ Therefore, the effectiveness of these two solutions for controlling bacterial biofilms seems to be equal.

In a study that evaluated fungal biofilm,^14^ a statistically significant reduction in the number of C. albicans hyphae (ClO2, P = 0.03; and CHX, P > 0.01) was observed upon treatment with ClO2 and CHX. Presence of C. albicans in hyphae in the oral mucosa indicated infection by this fungus.^29^ The antifungal effects of these two solutions have already been proven.^30^^,^^31^ In addition to reduction of hyphae, 60% of the patients treated with ClO2 and 70% of the patients treated with CHX were found to have achieved a cure for inflammation,^14^ which thus indicates the antifungal effects of ClO2 compared with those of CHX.

### Limitations

There were some limitations to this systematic review, given that in one study the concentrations of mouthwashes used in the experiment (CHX and ClO2) were not reported^17^ and in another the commercial name for the product (Fresh Chlor) was reported but the ClO2 concentration was not reported.^8^ In addition, no study addressed the expected adverse effects. Nor was it reported whether the results from each study were collected in a blinded manner. In this review, no meta-analysis could be performed, given the heterogeneity of purposes observed among the studies included. These conditions also make it difficult to generalize the conclusions, since the synthesis of the results was often based on a limited amount of evidence.

### Recommendations

Because the results from the mouthwashes assessed in this systematic review were equal to or more significant than those from the gold standard CHX,^8^^,^^11^^,^^12^^,^^13^^,^^14^^,^^16^^,^^17^ we recommend that future clinical and in vitro studies should be conducted; adverse effects should be considered at the time of evaluation in clinical studies; products should be specified; and blinding of results should be implemented and demonstrated.

## CONCLUSIONS

Mouthwashes containing PHMB and ClO2 are viable alternatives to CHX, since studies showed that the antimicrobial effects of PHMB were comparable with those of CHX and that the antimicrobial effects of ClO2 were even greater than those of CHX. These alternative solutions have little or no reported side effects or adverse effects. No study compared both PHMB and ClO2 with CHX.
